# Particle Size-Selective Assessment of Protection of European Standard FFP Respirators and Surgical Masks against Particles-Tested with Human Subjects

**DOI:** 10.1155/2016/8572493

**Published:** 2016-03-07

**Authors:** Shu-An Lee, Dong-Chir Hwang, He-Yi Li, Chieh-Fu Tsai, Chun-Wan Chen, Jen-Kun Chen

**Affiliations:** ^1^Department of Environmental Engineering and Science, Feng Chia University, Taichung 40724, Taiwan; ^2^Division of Occupational Hygiene, Institute of Labor, Occupational Safety and Health, Ministry of Labor, New Taipei City 22143, Taiwan; ^3^Institute of Biomedical Engineering and Nanomedicine, National Health Research Institutes, Miaoli 35053, Taiwan

## Abstract

This study was conducted to investigate the protection of disposable filtering half-facepiece respirators of different grades against particles between 0.093 and 1.61 *μ*m. A personal sampling system was used to particle size-selectively assess the protection of respirators. The results show that about 10.9% of FFP2 respirators and 28.2% of FFP3 respirators demonstrate assigned protection factors (APFs) below 10 and 20, which are the levels assigned for these respirators by the British Standard. On average, the protection factors of FFP respirators were 11.5 to 15.9 times greater than those of surgical masks. The minimum protection factors (PFs) were observed for particles between 0.263 and 0.384 *μ*m. No significant difference in PF results was found among FFP respirator categories and particle size. A strong association between fit factors and protection factors was found. The study indicates that FFP respirators may not achieve the expected protection level and the APFs may need to be revised for these classes of respirators.

## 1. Introduction

Respiratory protective devices (RPDs) are generally used to protect people from respiratory hazards, including chemical, biological, and radioactive materials. In the absence of engineering control and effective protection, RPDs can prevent workers in routine operations from life-threatening and health hazards. When RPDs cannot provide users with adequate protection, the risk of users' exposure to these respiratory hazards will increase and result in adverse health effects. Therefore, it is important to ensure that the RPDs provide adequate protection for users.

Disposable filtering half-facepiece respirators (DFHFRs), which are classified as air-purifying respirators, are widely used and accepted by workers in various industries and the general population. This is because DFHFRs are available in multiple sizes to fit a range of faces, are easy to maintain, offer little hindrance to wearers [1], and have the highest rating and evaluation in weight and convenience [2]. Among DFHFRs, NIOSH-approved N95 filtering facepiece respirators or higher are recommended for healthcare workers against airborne infectious diseases such as Ebola [3]. The US National Institute for Occupational Safety and Health (NIOSH) classifies particulate filtering facepiece respirators (FFRs) into nine categories (N95, N99, N100, P95, P99, P100, R95, R99, and R100) [4]. N (not resistant to oil) means that the respirators cannot be used in an oil droplet environment; R (somewhat resistant to oil) and P (strongly resistant to oil) mean that this respirator can be used for protection against nonoily and oily aerosols. Numerical designations 95, 99, and 100 show the filter's minimum filtration efficiency with 95%, 99%, and 99.97%, respectively.

The European Standard (EN 149:2001) classifies FFRs into three classes: FFP1, FFP2, and FFP3 with corresponding minimum filtration efficiencies of 80%, 94%, and 99%. Therefore, FFP2 respirators are approximately equivalent to N95 FFRs, making them recommended for use in the prevention of airborne infectious diseases in the US and some other countries. However, because FFP3 respirators provide the highest level of protection, they are the only FFP class acceptable to the Health and Safety Executive (HSE) for protection against infectious aerosols in healthcare settings in the UK [5]. This poses a question. Do respirators with higher filtration efficiencies provide greater protection when human subjects don the respirators?

Surgical masks (SMs) are used to block large particles (such as droplets, splashes, sprays, or splatter) that may contain microorganisms (e.g., viruses and bacteria) from reaching the nose and mouth. And although they are primarily intended to protect patients from healthcare workers by minimizing exposure of saliva and respiratory secretions to the patients, they generally do not form a tight seal against the face skin and so are not recommended to protect people from airborne infectious diseases. Therefore, SMs have been relegated for protection against infection through fluid repellence only. The protection provided by SMs against particles (0.04–1.3 *μ*m) is 8–12 times less than N95 FFRs [6], but they are both found to be equivalent for protection against influenza infection when the concentrations of infectious viruses are low [7]. SMs have been cleared by the Food and Drug Administration for sale in the US, while, in the UK, they must first comply with the Medical Devices Directive (MMD 93/42/EEC) [8] and be CE marked [9]. However, because N95/FFP2 respirators or above may be in short supply during a pandemic—or not available in many countries—it is important to know the protection efficiency of surgical masks.

The protection of respirators against microorganisms can be efficiently assessed by investigating respiratory protection against noninfectious particles of a corresponding particle size to infectious ones [10]. Therefore, the protection of the respirators is usually evaluated using sodium chloride (NaCl) and dioctyl phthalate (DOP) particles as challenge aerosols. NaCl particles are used to test filtration efficiencies against nonoily aerosols, while DOP particles are used for testing oily aerosols. N95 FFRs have been widely studied for filtration efficiency [11, 12], face seal leakage [12, 13], fit factors [14, 15], and protection factors [6, 10]. With respect to EN-specified FFP respirators, only the filtration efficiency was studied [16].

Because particles enter the respirator through face seal leaks and filter materials, respirator performance is assessed by fit testing, penetration testing, and total inward leakage (TIL) testing with human subjects. The TSI PortaCount® Plus with N95-Companion is commonly employed to quantify the respirator fit of N95 filtering facepiece respirators. However, the fit factor (FF) may not adequately reflect the true respiratory protection for a worker performing his/her actual work activities. As true workplace protection factors (WPFs) (during actual work activities) are often difficult to measure, NIOSH (2004) has proposed the use of the TIL test for assessing respirator performance as part of the certification process for a respirator [17]. The TIL test is meant to assess the protective level achieved by a respirator when contributions of all leakage paths are considered. Using either fit testing or filtration data to assess the overall respirator performance is not sufficient. The assessment of respiratory protection has frequently been conducted using the heads of mannequins [18] instead of human subjects, disregarding that human factors—such as facial dimensions [19] and breathing patterns and flow [13]—may interfere with protection provided by respirators.

This study aimed to investigate (1) the overall protection performance of disposable DFHFRs with different categories against particles of different sizes using the TIL test and (2) the relationship between protection factors and fit factors. We tested FFP1, FFP2, and FFP3 respirators from two manufacturers and three models of SMs to particle size-selectively assess respiratory protection with human subjects, because there is lack of information on the protection performance. A personal sampling system, which was modified according to the system developed by Lee et al. (2004) [20], was used to assess the protection provided by respirators. In order to control environmental factors, the experiment was conducted in a subject test chamber where the particle concentrations could be controlled. Furthermore, in order to particle size-selectively assess respiratory protection, this research carried out real-time particle measurement by using an electrical, low-pressure impactor (ELPI).

## 2. Methods

### 2.1. Description of the Personal Sampling System for Evaluation of Respiratory Protection

**Figure 1:**
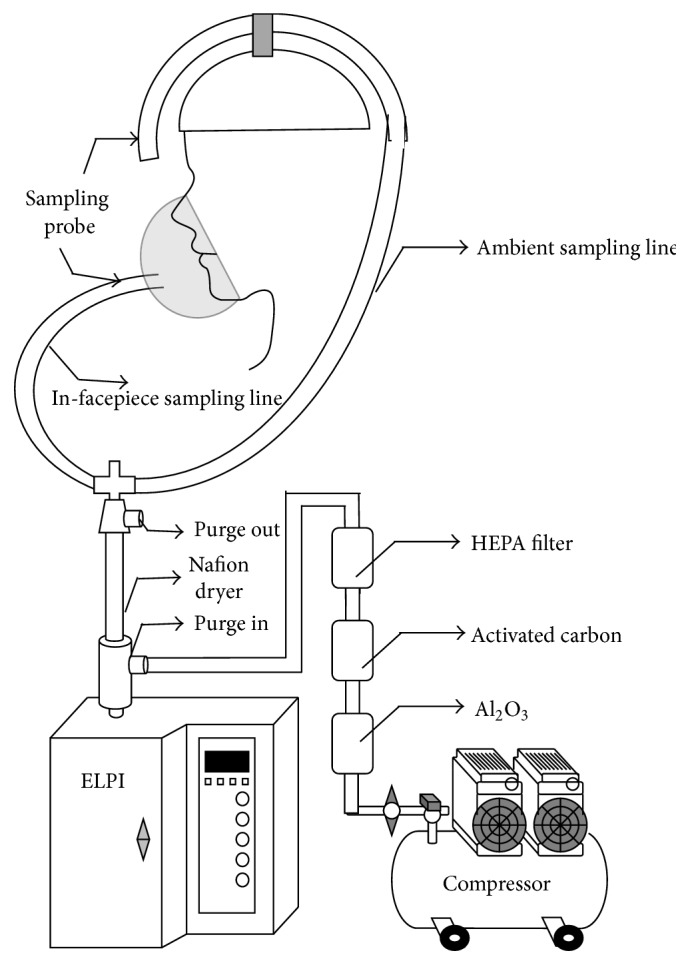
Personal sampling system for assessing respirator protection factors. This system was modified based on the system developed by Lee et al. (2008, 2004) [6, 20].

The assessment of the protection level of a respirator should collect samples from both outside and inside the respirator—the ratio of which is identified as the protection factor. Therefore, a personal sampling system assessing the protection of respirators requires two sampling lines (ambient and in-facepiece sampling lines) to collect samples outside and inside the respirators and also to connect the particle measuring equipment with the test respirator. In addition, because the sampling line inside the respirator contains nearly 100% relative humidity (RH)—which may affect particle measurement—the sampling system requires a dryer to reduce the moisture content of exhaled air. Based on the design concept, we modified the sampling system developed by Lee et al. (2008, 2004) [6, 20] to establish the system—presented in Figure 1. The sampling system had two sampling lines that collected the particles inside and outside the respirator. Each sampling line consisted of a sampling probe (adaptor kit 8025-N95, TSI Inc., St. Paul, MN, USA) and 1/2′′ Tygon tubing (Tygon tubing, Fisher Scientific, Pittsburgh, PA, USA), which were both connected with a three-way valve (Legris Inc., France), a Nafion dryer (HPMS PD-50T-12, Perma Pure LLC, NJ, USA), and an electrical low-pressure impactor (ELPI, Dekati Ltd., Finland). The sampling probe used in fit testing was used to collect samples inside the mask because it was easily mounted on the respirator's surface. A helmet with a copper tubing frame was used to fix two sampling lines to reduce face seal leaks due to the movement of the test system. The ambient sampling line was located on the top of the helmet, while the in-facepiece sampling line was placed above the shoulder. The length of the ambient sampling line was 136.9 cm, and the length of the in-facepiece sampling line was 122.6 cm. Two sampling lines were connected with the ELPI by using the three-way valve. The Nafion dryer was installed between the three-way valve and the ELPI to reduce to the RH of exhaled air down to 50–60%, which is about the same RH range as the indoor environment and can prevent bias in particle measurement. The additional dry air was introduced to create a humidity gradient during the operation of the Nafion dryer. This dry air was generated using a compressor equipped with an Al_2_O_3_ dehumidifier, activated carbon, and a HEPA filter (PN 12144, Pall Corporation, Bourne, MA, USA). The RH of the generated dry air was between 20% and 30%. In order to achieve efficient drying, the flow of dry air had to be 2-3 times greater than the sampling flow.

### 2.2. Establishment of the Subject Test Chamber

**Figure 2:**
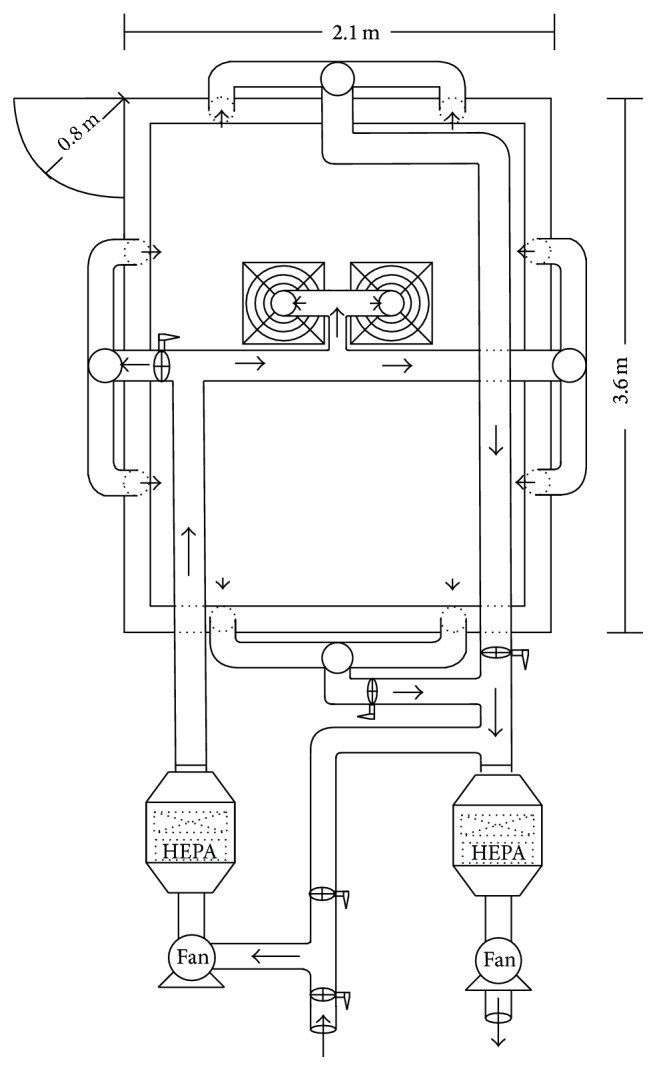
Schematic design of the subject test chamber.

Because the ELPI is costly, we only had one ELPI to serve two sampling lines. The stability of particle concentrations in the test environment was important when the PF was determined by a ratio of concentrations outside the respirator to those inside the respirator. The experiment was conducted in a subject test chamber with a ventilation system, which was located in the Laboratory for Industrial Hygiene and Safety in Feng Chia University in Taichung, Taiwan. The schematic design of the subject test chamber is shown in Figure 2. The total size of the subject test chamber was 19.5 m^3^ (3.6 m (length) × 2.1 m (width) × 2.58 m (height)), which represents the size of the typical residential room in Taiwan. The subject test chamber was enclosed with four vertical side walls and one ceiling, which were made of cleanroom aluminum honeycomb panels, and the floor was epoxy. An airtight door was installed to prevent contamination from outside of the chamber.

We used two high-pressure direct-drive blowers (1 HP, maximum airflow 27 m^3^/min, static pressure 24 mm H_2_O, TECO Electric & Machinery Co., Taipei, Taiwan) connected with HEPA air-purifying units to provide exhaust air and supply air to the subject test chamber. Each of the two HEPA air-purifying units consisted of a prefilter (35% filtration efficiency) and a HEPA filtration unit (99.97% filtration efficiency). The air was transported using 4- and 6-inch ducts. A total of 22 slot gates (14 gates for the air supply and 8 gates for the air exhaust) were installed on the vertical walls and ceiling of the test chamber. The left-side (airtight door side) and right-side walls had six supply gates: three pairs at 0.43, 1.29, and 2.15 meters, respectively, above the floor. The ceiling had two supply gates, which were located in the center of ceiling. The front wall, which was adjacent to the airtight door, had a pair of exhaust gates at 0.43 meters above the floor. The back wall had six exhaust gates, which were paired at each of the same three heights of two-side walls. Each slot gate could be operated independently of the others in the open or closed position. This allowed for the chamber to be operating using any possible combination of slots, thus creating various directions of air current in the chamber.

The air exchange rate could be controlled from 0 to 60 air exchanges per hour. The pressure of the test chamber was monitored in real time by a Magnehelic pressure gage (Model 2300–20MM, Dwyer Instrument, Inc., USA). The VelociCalc air velocity meter (Model 9535, TSI Inc., USA) and the inclined-vertical manometer (Model MARK II MM-80, Dwyer Instrument, Inc., USA) measured the velocity of the slot gates and the pressure drop of the HEPA air-purifying unit, in order to evaluate the air exchange rate of the test chamber and the performance of the HEPA air-purifying unit. The relative humidity and temperature of the test chamber could be maintained at 25 ± 3°C and 40–60% by the air conditioner and dehumidifier.

### 2.3. Aerosol Generation in the Subject Test Chamber

NaCl solution (1.5 g/100 mL) was aerosolized in the test chamber by a six-hole Collison nebulizer (BGI Inc., Waltham, MA, USA). A flow rate of 12 L/min of NaCl particle-laden air was then mixed with a 16 L/min of diluted dry air. Since laboratory-generated particles might carry high electrical charges, the entire airflow of 28 L/min was directed through a charge equilibrator (3.6 *μ*Ci Am 241) to achieve the Boltzmann charge equilibrium. An air circulation fan (with a flow rate of about 900 CFM) located at the outlet of the aerosol generation system distributed the aerosolized particles within the test chamber. The particle concentrations inside the test chamber could be stably controlled at (0.1~1) × 10^5^ particles/cm^3^.

### 2.4. Requirement and Characteristics of Human Subjects

To test the respirators and surgical masks, 30 students aged 18 to 24 were recruited from Feng Chia University (15 men and 15 women). Values for the distance from the Menton process to the top of the head were 13.0 to 25.6 cm (21.8 ± 2.4 cm); for the bitragion breadth, they were 12.5 to 16.2 cm (14.4 ± 1.0 cm); and for the lip width, they were 3.7 to 6.0 cm (4.8 ± 0.5 cm). All subjects were nonsmokers and inexperienced respirator users. Human testing in this study had been approved by the Institutional Review Board of China Medical University Hospital, Taichung, Taiwan, through the approval number DMR99-IRB-165—and each test subject provided written informed consent. A researcher informed the subjects that they could demand suspension of the experiment if they experienced any discomfort.

For the total inward leakage (TIL) test carried out in the laboratory, each subject had to be free of allergies and any cardiovascular or respiratory tract diseases. Each subject was not permitted to drink or smoke half an hour before the test, and male subjects were required to be clean-shaven [21]. A researcher taught subjects how to properly don the respirator and implemented the fit testing. All participants had to undergo the fit test before the TIL experiment was carried out.

### 2.5. Selection of the FFP Respirators and Surgical Masks

In order to evaluate the protection performance of similar DFHFR (the same appearance, shape, materials, etc.) with different categories against particles of different sizes, we selected cone shaped FFP1, FFP2, and FFP3 respirators from two different companies (A and B represented two companies in text, tables, and figures), comprising the same classes of respirators and one single size, yet with different filtration efficiencies. In addition, flat shaped surgical masks—C, D, and E—from three different manufacturers were chosen for testing.

### 2.6. Experimental Protocol

Before the fit testing and TIL test, each subject was trained to wear the tested respirator by guidance from a researcher. A user seal check was performed to ensure that an adequate seal was achieved when the respirator was put on the subject's face. Subjects performed the US's Occupational Health and Safety Association's (OSHA) fit testing exercises, including normal breathing, deep breathing, turning head side to side, moving head up and down, talking, grimace, bending over, and returning to normal breathing [21]. The fit factor for each exercise was recorded using a PortaCount Plus (TSI Inc., St. Paul, MN, USA). The overall fit factor (FF) is calculated as follows:

(1) FF=Number  of  exercises1/ff1+1/ff2+1/ff3+1/ff4+1/ff5+1/ff6+1/ff7+1/ff8,

where ff_1_, ff_2_, ff_3_, and so forth are the fit factors for Exercises 1, 2, 3, and so forth (grimace (Exercise #6) is excluded).

For the TIL test, the numerical concentrations of NaCl particles were size-selectively measured using the ELPI with a sampling flow of 10 L/min. The ELPI measures the numerical concentration of particles in an aerodynamic size, ranging from *D*_*a*_ = 0.028 to 10.01 *μ*m, in 12 channels. In this study, we used six channels with geometric mean (GM) diameters of 0.121, 0.203, 0.318, 0.486, 0.766, and 1.238 *μ*m, in the particle size range of 0.093–1.61 *μ*m. Particle sizes between 0.093 and 1.61 *μ*m were measured because viral and bacterial particles fall within this range. When the particle concentrations were stable in the test chamber, the subjects equipped with the personal system donned the test respirators and performed the OSHA fit testing exercises. Each exercise was performed for two minutes and the particle concentrations inside the respirator were averaged over the second minute. The concentration inside the respirator (*C*_in_) for the entire test was averaged over all the exercises, excluding the grimace maneuver. The particle concentrations outside the respirator (*C*_out_) were measured before and after the subject performed the exercises. The average of these concentrations was used as the concentration outside the respirator for each test. The protection factor (PF) was calculated by dividing the particle concentrations outside the respirator with those inside the respirator:

(2) $$\begin{array}{c} PF=\frac{C_{out}}{C_{in}}. \end{array}$$

Each subject was tested on the FFP1, FFP2, and FFP3 respirators of manufacturer A and manufacturer B and on the three models of surgical masks (C, D, and E).

The particle losses in the sampling line have been addressed in our previous study [20], where we found that a difference in the penetration efficiencies of particles between the two sampling lines was due to slightly different configurations. Therefore, all PFs presented in this paper were corrected by a ratio of concentrations measured in the two sampling lines when no respirator was attached in the system. These ratios varied from 0.97 to 1.04, depending on the particle size.

### 2.7. Data Analysis

Data were organized and managed using Microsoft Excel 2013, and the plots were made by SigmaPlot 10.0. The data analysis was performed using ANOVA test, *t*-test, and the Pearson correlation model provided by SPSS 12.0 for Windows (SPSS Inc., USA) software. All data were log-transformed before conducting statistical testing. *p* values of <0.05 were considered significant. The difference in fit factors and PFs among FFP respirators was examined by the ANOVA test followed by a pairwise comparison using Tukey's Studentized Range test. This test method was also used to examine the effect of particle size on PFs. The Pearson correlation coefficients were obtained to examine the association between protection factors and fit factors.

## 3. Results

### 3.1. Fit Testing Results

Before we carried out human tests to assess actual respirator performance, the subjects were first required to undergo quantitative fit testing that was conducted using a PortaCount Plus, not the N95 Companion. There are three reasons for not using the N95 Companion in this study: (1) Generally, only FFP2 respirators use the N95 Companion for fit testing, whereas this type of mask fit tester is unnecessary in conducting fit tests for FFP respirators and surgical masks—which are the focus of this study. (2) The original concept developed by the N95 Companion for fit testing respirators is to measure the amount of particulate getting through the face seal only. However, Rengasamy et al. (2012) have shown that negatively charged particles between 0.04 and 0.06 *μ*m are small enough to penetrate not only the face seals of these respirators, but also their filters [22]—ultimately skewing test results and making this type of fit testing inaccurate. (3) In order to establish the relationship between PFs and fit factors in assessing protection against particles, the wider-sized range of the PortaCount Plus covering the size of tested particles was favorable and comparable. The fit testing results are shown in Table 1. The highest percentage of subjects passing FFP fit testing was 93.3% for FFP1_A and FFP2_B. No subjects passed fit testing with SMs. The nine respirators are listed in descending order of the geometric mean as follows: FFP2_B (174.2) > FFP1_A (171.6) > FFP3_B (138.4) > FFP3_B (112.4) > FFP3_A (103.2) > FFP1_B (138.4) > SM_E (3.1) > SM_C (3.0) > SM_D (2.1).

### 3.2. Protection Factors of Tested Respirators

**Figure 3:**
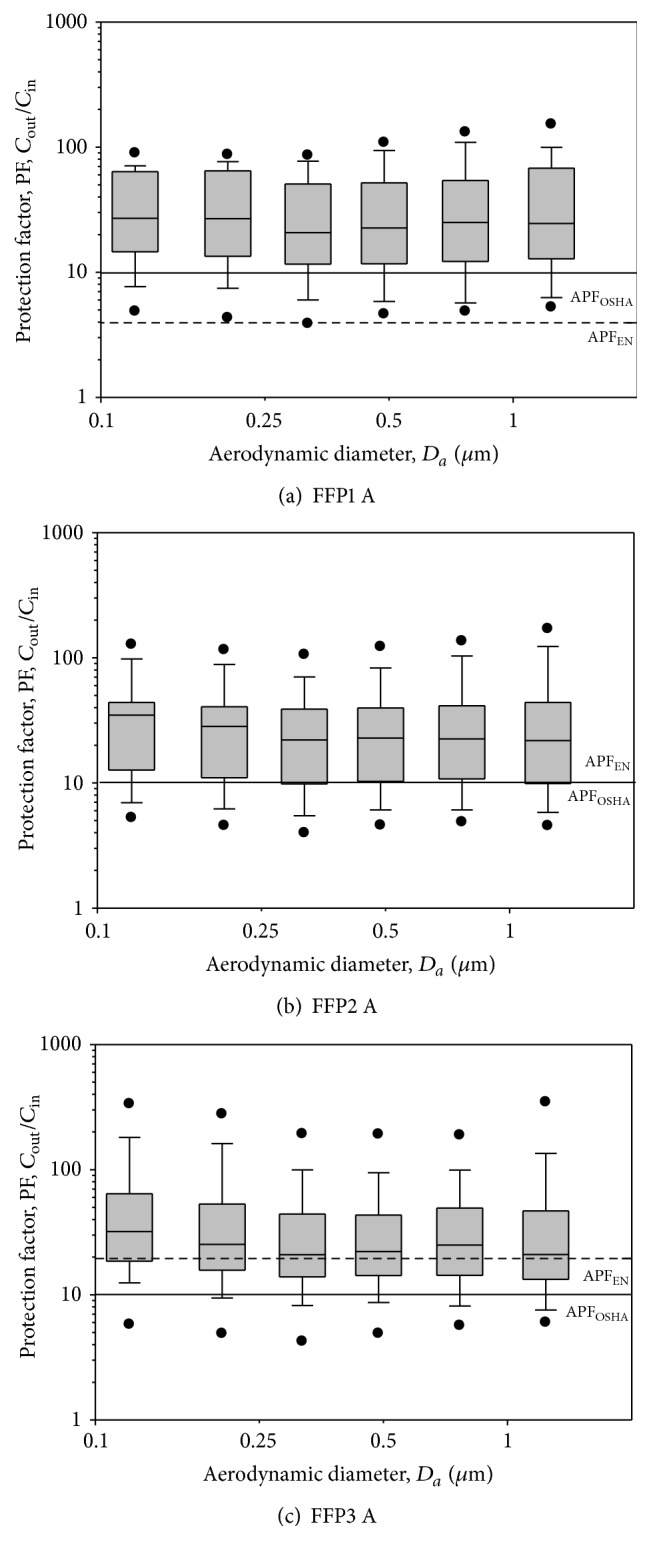
PF-values against particles in size range of 0.093–1.61 *μ*m for FFP1, FFP2, and FFP3 respirators manufactured by company A. The tests were performed when the FFP respirators were donned on human subjects. Total observations are 30 (30 subjects). The boxplots show the following: dots (from bottom) represent 5% and 95% percentiles; horizontal lines (from bottom) represent 10%, 25%, 50%, 75%, and 90% percentiles. APF refers to the assigned protection factor for that particular mask.

**Figure 4 fig4:**
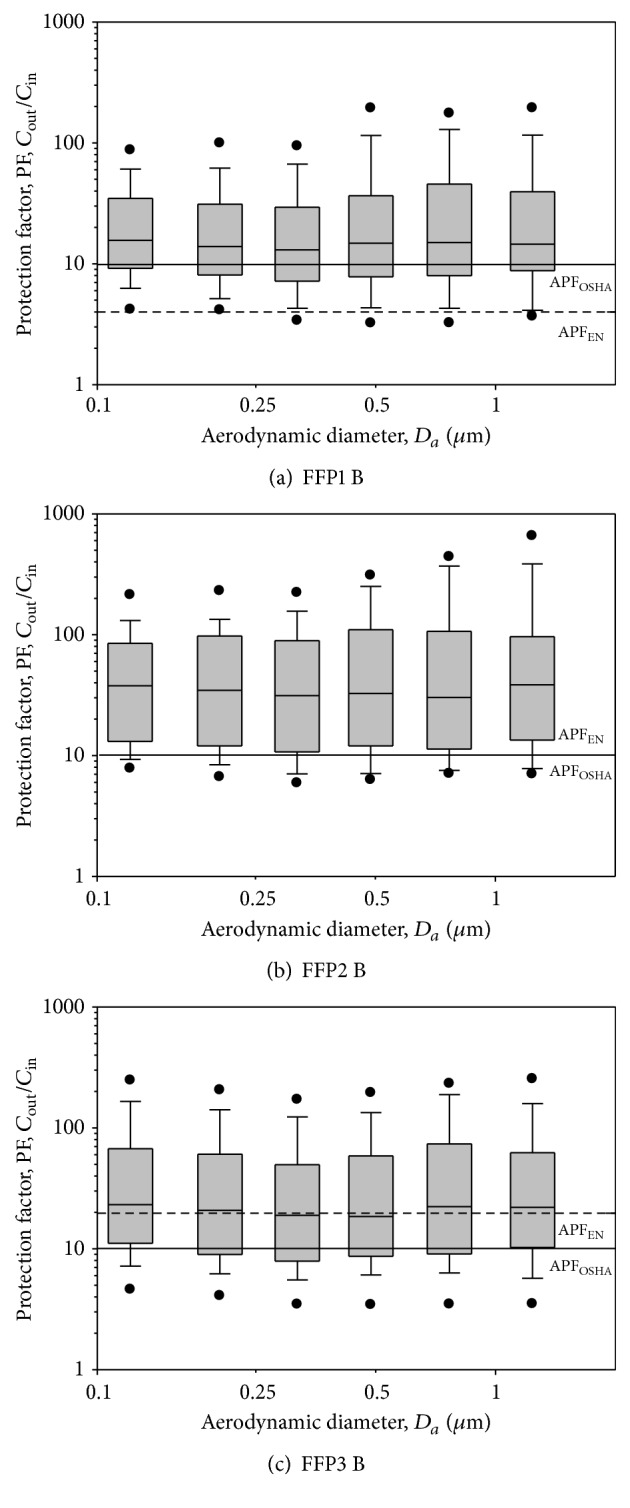
PF-values against particles in size range of 0.093–1.61 *μ*m for FFP1, FFP2, and FFP3 respirators manufactured by company B. The tests were performed when the FFP respirators were donned on human subjects. Total observations are 30 (30 subjects). The boxplots show the same as in Figure 3. AFF refers to the assigned protection factor for that particular mask.

**Figure 5:**
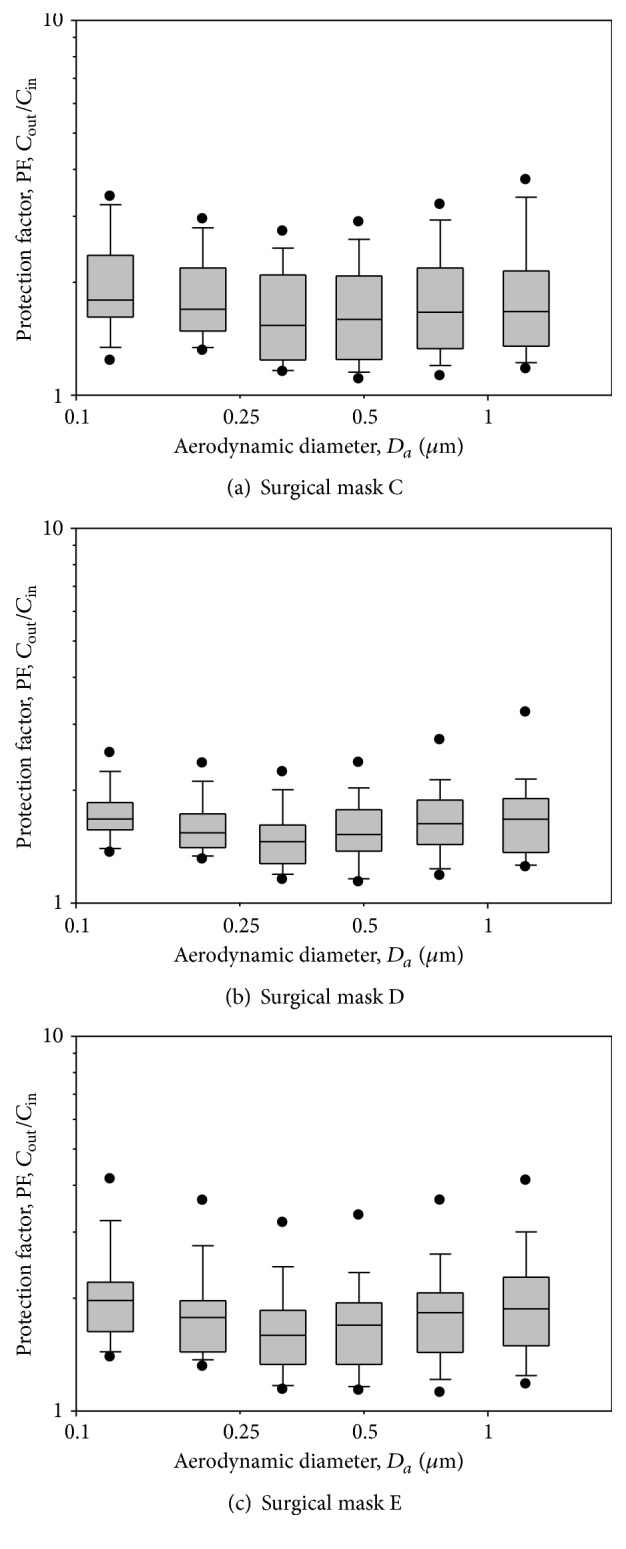
PF-values against particles in size range of 0.093–1.61 *μ*m for three models of surgical masks: C, D, and E. The boxplots show the same as in Figure 3. The tests were performed when the surgical masks were donned on human subjects. Total observations are 30 (30 subjects).

After fit testing, all 30 subjects underwent respirator and surgical mask testing. Each subject was required to don nine DFHFRs to assess the protection of the respirators. The experimental results were plotted for PFs versus particle size in a boxplot chart. The results of nine DFHFRs are presented in Figures 3 [Fig fig4]–5 and Table 2. Figures 3 and 4 show the PFs of FFP respirators, respectively, for manufacturers A and B in the particle size range of 0.093–1.61 *μ*m and represent the range of viral and bacterial sizes. Regardless of the fit testing results, the overall GM of PFs was 19.6 for PPF1 respirators, 27.1 for FFP2 respirators, and 26.7 for FFP3 respirators. The respective GMs for subjects passing the fit testing were 25.5, 30.9, and 37.6. Thus, on average, the PFs were 1.1–1.4 (30.9/27.1–37.6/26.7) times greater when only data for those who passed the fit testing were included. The differences were not statistically significant (*p* > 0.05), nor were the PFs statistically significantly different among FFP classes (*p* > 0.05) for both data sets (all subjects and subjects passing the fit testing). The lowest PFs occurred between particle sizes of 0.263 and 0.384 *μ*m. PFs were not significantly different among different particle sizes in the size range of 0.093–1.61 *μ*m (*p* > 0.05) for both data sets (all subjects and subjects passing the fit testing). The assigned protection factor (APF) of 10 for N95 FFRs [23] is shown by a horizontal solid line, denoted as 4, 10, and 20, respectively, for FFP1, FFP2, and FFP3 respirators (which are represented by a short dashed line in the figures). The APF value represents the level of protection that a properly functioning respirator is expected to provide for adequately fitted and trained users in a respiratory protection program in the workplace. Among the 60 tested FFP1 respirators (30 subjects × 2 manufacturers), PFs below 4, 10, and 20 were found for 5.0%, 28.3%, and 53.3% of the respirators, respectively. The respective percentages for PFs of FFP2 respirators were 1.7%, 18.3%, and 35.0%, while they were 1.7%, 20.0%, and 41.7% for FFP3 respirators. The respective percentages for subjects passing the fit testing were 2.5%, 17.5%, and 40.0% for FFP1 respirators, 0.0%, 10.9%, and 30.4% for FFP2 respirators, and 0.0%, 12.8%, and 28.2% for FFP3 respirators. The percentages significantly decreased after fit testing (*p* < 0.05).

PFs for the three models of SMs are shown in Figure 5 and Table 2. The overall geometric mean of PFs was 1.7. The minimum numbers of the PFs were also found to be approximately between 0.263 and 0.384 *μ*m particle size for SMs C, D, and E. It was found that PFs were not significantly different among different particle sizes in the size range of 0.093–1.61 *μ*m (*p* > 0.05) for SMs C and E, while the particle size was found to significantly affect PFs for SM D. We found that PFs for particles > 0.616 *μ*m were significantly greater than the minimum PFs for SM D (*p* < 0.05). None of the tested SMs had PFs > 4. With respect to the geometric mean of PFs, the overall PF provided by SMs was 11.5 times less than that for FFP1 respirators, 15.9 times less than that for FFP2 respirators, and 15.7 times less than that for FFP3 respirators (*p* < 0.05).

### 3.3. Association between Fit Factors and Protection Factors

**Figure 6:**
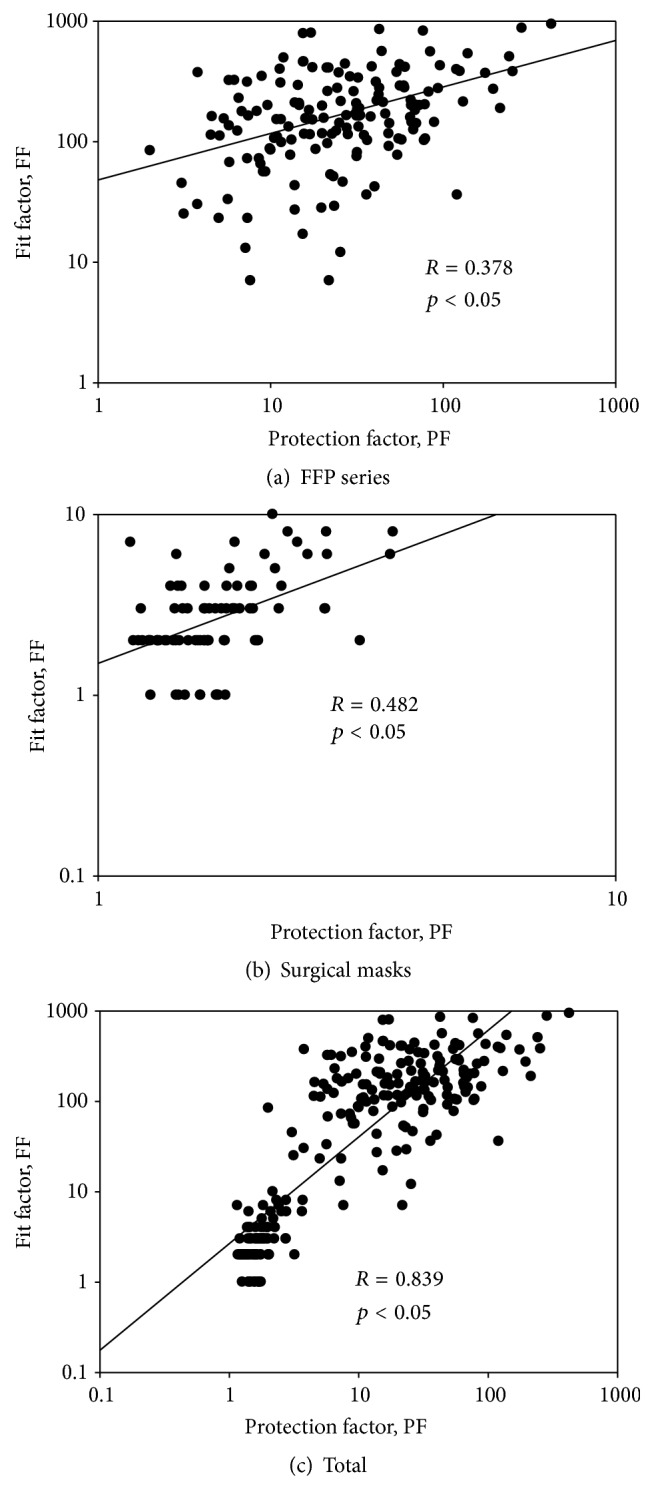
The association between fit factors and protection factors. The associations for FFP respirators, surgical masks, and total (all respirators combined) are presented, respectively, in (a), (b), and (c).

Figure 6 presents the regression plots for the associations between fit factors and PFs. We found that the correlation coefficients were 0.378 for FFP respirators and 0.482 for SMs. These values were in the middle of weak (*r* = 0.3) and moderate (*r* = 0.5) positive linear relationships and were statistically significant (*p* < 0.05). When the data for FFP respirators and SMs were combined, the correlation coefficient increased to 0.835 (*p* < 0.05), which presented a significantly strong positive association between fit factors and PFs.

## 4. Discussion

Current guidance issued by the Centers for Disease Control and Prevention (CDC) and the Health and Safety Executive (HSE) recommends the use of N95 or higher respirators and FFP3 respirators against airborne infectious diseases in healthcare settings. When these certified DHFFRs are in short supply or not available, SMs may be an alternative. No previous investigations have utilized human subjects to investigate the protection provided by FFP respirators against particles in the size of 0.093–1.61 *μ*m, representing bacterial and viral size ranges.

We found the minimum PF for both FFP respirators and SMs appeared for particle sizes between 0.263 and 0.384 *μ*m. When particles pass through face seal leaks and filter materials, diffusion causes deposition of smaller particles on the surface, while impaction and interception dominate deposition of larger particles. This is why we had minimum protection for particle sizes between 0.263 and 0.384 *μ*m. However, the protection provided by both FFP respirators and SMs was not significantly affected by the particle size ranging from 0.093 to 1.61 *μ*m. In a recent study [24], we found that particle penetration through face seal leaks was greater than that through filter material (SMs were ~4 to 8 times greater, and FFP respirators were ~1.5–6.7 times greater). This finding is similar to the results published by Grinshpun et al. (2009) for N95 FFRs and SMs [12]. The subjects' average breathing rate measured was between 8.4 and 16.9 L/min during fit testing exercises. The leak flow through face seal leaks was laminar at these flow rates [25], resulting in PFs independent of particle size. The size ranges of viral and bacterial particles fall into this size range, and they are expected to have similar PFs. In contrast, Lee et al. (2008) and Grinshpun et al. (2009) show that PFs are significantly size-dependent for N95 filtering facepiece respirators in a similar size range [6, 12]. However, Grinshpun et al. (2009) have found that the particle penetration through face seal leaks is not significantly size-dependent [12]. Our recent study has shown that face seal leaks contribute more particle concentrations inside the respirator than does filter penetration [24] and indicates that characteristics of face seal leaks (including shape, size, etc.) might affect particle penetration inside the respirator, resulting in size-independent PFs.

For subjects passing the fit testing, the percentage of the respirators that had PFs greater than 4 was greater than 95% for FFP respirators. However, the corresponding percentages for PFs greater than 10 and 20, respectively, were 82.5% and 60.0% for FFP1 respirators, 89.1% and 69.6% for FFP2 respirators, and 87.2% and 71.8% for FFP3 respirators. This is interesting in that the FFP3 respirators performed the same or worse than the FFP2 respirators. This also happened for the fit testing results where subjects donning FFP3 respirators did not have the highest fit testing pass rates, which indicates that respirators with the highest filtration efficiencies are not necessary for having the best fit factors and PFs. FFP3 respirators generally have a greater packing density and pressure drop in respirator filters than do FFP2 respirators, which results in a more pronounced particle penetration through face leaks than with filter materials. Face seal leaks do not perform like filters to prevent particles from entering the respirator, which results in FFP3 respirators not being equal to or better than FFP2 respirators, as was expected. This study also shows that the PFs for FFP respirators increased when subjects who did not pass the fit testing were excluded from the analysis, which further demonstrates the power of fit testing to improve respirator protection.

The APF is the ratio of pollutant outside the device to that inside the device and is defined by British Standard BS EN 529:2005 as the level of respiratory protection that can realistically be expected to be achieved in the workplace by 95% of adequately trained and supervised wearers using a properly functioning and correctly fitted respiratory protective device and is based on the 5th percentile of the Workplace Protection Factor (WPF) data. In the US, the APF is 10 for half masks, while they are 4, 10, and 20, respectively, for FFP1, FFP2, and FFP3 respirators in the UK. The fifth percentile of PFs obtained in this study was 5.3 for FFP1 respirators, 6.7 for FFP2 respirators, and 6.1 for FFP3 respirators. Only the APF result for FFP1 respirators fit the European standard, indicating that APF standards for FFP2 and FFP3 respirators should be considered for revision. This finding is similar to previous results for N95 filtering facepiece respirators [6, 10, 26, 27]. In addition, the fifth percentile values of FFP respirators were close to each other, whereas the APF standard for FFP respirators could be one number. Based on the results obtained in our study, we recommend the APF value of 5 for FFP respirators. Although there were no significant difference in PFs among FFP respirators, FFP2 respirators and above are recommended in healthcare settings against infectious diseases. This is because the overall performance of FFP1 respirators is somewhat inferior to FFP2 and FFP3 respirators. Regardless of fit testing, the overall PF provided by SMs was, significantly, 11.5 times less than that for FFP1 respirators, 15.9 times less than that for FFP2 respirators, and 15.7 times less than that for FFP3 respirators. This suggests that SMs are not a good substitute for FFP respirators when concerns exist about airborne transmission of viral or bacterial pathogens.

For tight-fitting respirators with higher filtration efficiencies such as N95 respirators and FFP respirators, Coffey et al. (2004) found that subjects failing a fit test also received adequate protection, resulting in high alpha errors [26]. High alpha errors could result in subjects erroneously failing a particular fit test with a higher level of protection than those subjects correctly passing the fit test. For loose-fitting respirators with lower filtration efficiencies, the FFs and PFs are too small to demarcate any difference detected by the measurement devices, which is why we had weak association between FFs and PFs for FFP respirators and SMs. However, the difference in FFs and PFs between FFP respirators and SMs is very large. Therefore, we had greater FFs corresponding to greater PFs for FFP respirators, while we had smaller FFs corresponding to smaller PFs for SMs. This resulted in an increase in the *r* value when the two data were combined. Zhuang et al. (2003) also found that the association between PFs and FFs decreased when subjects who failed the fit testing were excluded but increased when all subjects were included in the analysis [28]. Although the fit factors are meant to assess the face seal leaks rather than overall leaks evaluated by the PFs, our results indicate that the PFs of respirators against particles can be assessed by fit factors. Considerable time, labor, and expense could therefore be saved by assessing PFs of respirators against particles. However, there are clear differences in FFP respirators and surgical masks, and SMs do not fall under OSHA fit test requirements. It is recommended that the protection of FFP respirators and SMs should be investigated separately when fit factors are used to assess actual respirator performance.

The study was conducted in a test chamber instead of a real work environment. Due to restrictions within the test environment, the data interpretation extending to other work environments may be limited. Also, because the subjects were selected only from a younger population in Taiwan and the number of subjects was small, there is a limitation for data interpretation extending to all workers and other races. In addition, we only tested FFP1, FFP2, and FFP3 respirators manufactured by two companies, and three models of SMs. More respirators are needed for further studies to confirm our results.

## 5. Conclusion

The tested FFP respirators and SMs in this study were observed to have the worst protection against particles between 0.263 and 0.384 *μ*m. The protection factors of FFP respirators against particles in the size range of 0.093–1.61 *μ*m were not size dependent. The size ranges of viral and bacterial particles fall into this size range, and they are expected to have similar PFs. The FFP respirators provided about 11.5 to 15.9 times better protection than the SMs, suggesting that SMs are not a good substitute for FFP respirators when concerns exist about airborne transmission of bacterial and viral pathogens. About 18.3% of the tested FFP2 respirators had PFs <10, and ~41.7% of the tested FFP3 respirators had PFs <20, indicating that the European standard for APF of 10 for FFP2 respirators and 20 for FFP3 respirators may overestimate the actual protection offered by these respirators against particles in the size range of 0.093–1.61 *μ*m. PFs among FFP respirator classes were not significantly different, indicating the APF value for FFP respirators could possibly be revised to one value rather than three for FFP1, FFP2, and FFP3 respirators. Fit factors could be used as an indicator to assess the protection provided by FFP respirators and SMs.

## Figures and Tables

**Table 1:** Fit factors of 6 FFP respirators and 3 surgical masks (SMs). Total observations are 30 (30 subjects).

	FFP1_A	FFP2_A	FFP3_A	FFP1_B	FFP2_B	FFP3_B	SM_C	SM_D	SM_E
Percentage of fit factors > 100 (%)	93.3	80.0	73.3	60.0	93.3	76.7	0	0	0
Geometric mean	171.6	112.4	103.2	75.0	174.2	138.4	3.0	2.1	3.1
Geometric standard deviation	1.7	2.2	3.0	2.9	2.5	4.9	1.7	1.6	1.8

**Table 2:** Protection factors of 6 FFP respirators and 3 surgical masks (SMs). Total observations are 30 (30 subjects).

	FFP1_A	FFP2_A	FFP3_A	FFP1_B	FFP2_B	FFP3_B	SM_C	SM_D	SM_E	Total FFP1	Total FFP2	Total FFP3	Total SM
The fifth percentile^*∗*^	5.0	6.5	13.5	6.4	8.1	6.1	1.2	1.3	1.3	5.3	6.7	6.1	1.2

GM ± GSD (all subjects)	24.1 ± 2.5	23.4 ± 2.5	31.9 ± 3.0	15.9 ± 2.4	31.4 ± 2.8	22.6 ± 3.1	1.7 ± 1.3	1.5 ± 1.2	1.8 ± 1.3	19.6 ± 2.5	27.1 ± 2.7	26.7 ± 3.1	1.7 ± 1.3

GM ± GSD (pass fit test)	26.6 ± 2.5	30.0 ± 2.3	46.5 ± 3.1	23.7 ± 2.4	31.6 ± 2.7	30.8 ± 3.0				25.5 ± 2.5	30.9 ± 2.5	37.6 ± 3.1	

^*∗*^The fifth percentile was calculated only for subjects passing the fit test with FFP respirators. Because no subjects passed the fit test for surgical masks, the fifth percentile was calculated for all subjects wearing surgical masks (*n* = 30). GM refers to the geometric mean of PF data; GSD refers to the geometric standard deviation of PF data.
